# REHOSPITALIZATION RISK AMONG VETERANS RECEIVING POST-ACUTE HOME HEALTH SERVICES: DIFFERENCES BY PAYOR SOURCE

**DOI:** 10.1093/geroni/igae098.3889

**Published:** 2024-12-31

**Authors:** Ellen McCreedy, Frank Devone, Pedro Gozalo, Stefan Gravenstein, Marguerite Daus, Christine Jones, Kate Magid, Kali Thomas

**Affiliations:** Veterans’ Administration, Providence, Rhode Island, United States; United States Department of Veterans Affairs, Providence, Rhode Island, United States; Veterans’ Administration, Providence, Rhode Island, United States; United States Department of Veterans Affairs, Providence, Rhode Island, United States; Department of Veterans Affairs, Aurora, Colorado, United States; VA Eastern Colorado Healthcare System, Aurora, Colorado, United States; Rocky Mountain Regional VA, Aurora, Colorado, United States; Johns Hopkins University, Baltimore, Maryland, United States

## Abstract

Medicare-eligible Veterans who require post-acute skilled home health (HH) after a hospitalization can have HH paid for by the Veterans’ Administration (VA) or Medicare. Our prior work showed Veterans enrolled in Medicare Advantage (MA) are more likely to have the VA pay for HH than MA. In this analysis, we look at differences in 90-day rehospitalization among MA-enrolled Veterans receiving VA-paid post-acute HH versus MA-paid post-acute HH. All Veterans enrolled in MA and receiving HH after an index hospitalization at a VA medical center between October 1, 2016 and September 30, 2019 were included. Data were extracted from the VA electronic medical record. We used inverse probability weighting to address baseline differences in age, patient complexity, and past utilization. We used cox proportional hazards models to estimate the effect of VA- versus MA-paid HH on the risk of 90-day rehospitalization. There were 14,501 eligible hospitalizations requiring HH; 8,753 (60.4%) received VA-paid HH, and 5,748 (39.6%) received MA-paid HH. On average, Veterans receiving VA-paid HH waited 3.7 days (SD: 3.2) to start HH, compared to 4.8 days (SD: 3.8) for Veterans receiving MA-paid HH. After preliminary weighting, Veterans who received VA-paid HH had a lower risk of 90-day rehospitalization, compared to Veterans who received MA-paid HH (adjusted hazard ratio=0.88; 95% CI: 0.83,0.93). Veterans whose HH was paid for by MA received services one day later, on average, and had higher risk of 90-day rehospitalization compared to Veterans receiving VA-paid HH. Potential implications on quality of post-acute care for MA-enrolled Veterans discussed.

